# Evaluation of accuracy of *plain* radiography in determining the Risser stage and identification of common sources of errors

**DOI:** 10.1186/s13018-014-0101-8

**Published:** 2014-11-19

**Authors:** Jae Hyuk Yang, Amit Wasudeo Bhandarkar, Seung Woo Suh, Jae Young Hong, Jin Ho Hwang, Chang Hwa Ham

**Affiliations:** Department of Orthopedics, Scoliosis Research Institute, Korea University Guro Hospital, Guro, Korea; Department of Orthopedics, Korea University Ansan Hospital, Guro, Korea; Division of Pediatric Orthopaedics, Orthopaedic Surgery, Yonsei University College of Medicine, Severance Children’s Hospital, Seoul, Korea

**Keywords:** Pelvis, Ossification, Risser’s sign, Computed tomography, Concordance rate

## Abstract

**Background:**

Risser’s sign is *an established* radiological marker for predicting *growth potential* in adolescent idiopathic scoliosis, *but the accuracy of Risser’s staging has been debated. This research was designed to evaluate the accuracy of Risser’s staging and to identify causes of errors in Risser’s staging.*

**Materials and methods:**

Plain radiographs of 89 adolescent idiopathic scoliosis patients were evaluated for Risser’s stage using both the Original and French methods. A three-dimensional computed tomography (3D-CT) was used to evaluate the accuracy of the plain radiographs. Inter- and intra-observer reliability of both methods was assessed on radiographs and 3D-CT images using weighted kappa statistics. The concordance rate for Risser’s staging between plain radiographs and 3D-CT images were calculated. The various sources of staging differences between the two imaging methods were noted, grouped, and analyzed to identify common error patterns.

**Results:**

Intra- and inter-observer staging reliabilities on plain radiography were 0.91 and 0.94, respectively, using the Original method and 0.91 and 0.92, respectively, using the French method. Intra- and inter-observer reliabilities on 3D-CT were 0.98 and 0.99, respectively, using the Original method and 0.97 and 0.99, respectively, using the French method. Mean concordance rates between plain radiography and 3D-CT were 59.76% and 67.42% using the Original and French methods, respectively. Common sources of error leading to misinterpretation of Risser’s staging were miscalculation of apophysis excursion, skip ossification, isolated *non-linear* ossification, micro-fusion, and pseudo-fusion.

**Conclusions:**

Risser’s staging by plain radiography is reliable but not accurate. Variations in the iliac apophysis ossification and misinterpretation of apophysis fusion are the main sources of error.

## Introduction

In 1936, Joseph C. Risser described the capping and progression of iliac apophysis ossification as an invaluable aid in determining spinal skeletal maturity [1]. As there is a strong correlation between vertebral column growth and the length of iliac apophyseal ossification, excursion, and fusion, Risser’s sign became an *established* radiological marker for predicting curve progression in adolescent idiopathic scoliosis (AIS) patients [1]. Commonly, only an anteroposterior pelvic radiograph is assessed to calculate Risser’s stage of an individual; however, the reliability and accuracy of Risser’s staging by *plain* radiography (*PR*) have been questioned [2]. *Understanding that a strong correlation exists between skeletal growth and the sequence of iliac apophyseal ossification, we hypothesized that the common Risser staging method involving PR is not accurate and often results in miscalculation of the iliac apophyseal excursion, ossification, and fusion due to superimposition of the iliac bone and apophysis.* To overcome the assumed limitations, some authors have suggested the inclusion of additional views of the ilium for improved assessment of the iliac apophysis; however, despite these new studies, the reliability and accuracy of Risser’s staging remain inconsistent and controversial [3-6]. Three-dimensional computed tomography (3D-CT), which overcomes all of the limitations of two-dimensional imaging, is undoubtedly the best tool to assess actual changes in the iliac apophysis and may be the best tool to determine the accuracy of Risser’s staging [7-9]. We utilized 3D-CT to evaluate the accuracy of Risser’s staging performed using *PR* in a homogenous population of AIS patients. An added objective was to identify common patterns and causes of error in the interpretation of iliac apophysis excursion by *PR and to identify improvements to Risser’s staging system using PR.*

## Materials and methods

After obtaining the approval from the Institutional Review Board, a retrospective review of patients who underwent corrective surgery for idiopathic scoliosis between 2004 and 2009 was performed. As a routine scoliosis assessment, these patients subjected to a series of standard whole spine radiographs. As part of our standard institutional protocol, whole spine radiographs were acquired on a long cassette with the patient in an erect position so that the iliac crest apophysis could be visualized completely. The 3D-CT was not performed routinely but was offered to the patients as an option for improved preoperative planning. Between 2004 and 2009, 3D-CT scanning was used for preoperative planning in the 89 patients that were included in our study. Before 3D-CT evaluation, all patients were informed of the risk of radiation exposure, and all patients provided informed consent.

A 3D-CT evaluation was performed with a 16-channel instrument (Somatom Sensation 16, Siemens AG, Erlangen, Germany) using the following protocol: 120 kV, 80 mAs, 5.63 CTDI_vol_, 432 DLP, and CAREDOSE modulation. Axial cuts were acquired at 2 mm intervals from the cervical spine to the pelvis and were used in reconstruction of the coronal, sagittal, and three-dimensional views of the whole spine and pelvis [8]. Coronal sections reconstructed at 2 mm intervals were also used to evaluate the shape of the deformity, size of the pedicle, and the fusion of apophysis in greater detail [8].

### Risser’s staging

Two fellowship-trained orthopedic spinal surgeons evaluated Risser’s staging on *PR* and 3D-CT scans using the Original method (*OM*) and the French method *(FM)* [10]. In the *OM* of Risser’s staging, which is practiced in the United States, the iliac wing is divided into the following six grades: Risser 0, no ossification; Risser 1, ossification within the first quarter of the crest (up to 25%); Risser 2, ossification extending into the second quarter of the crest (25%–50%); Risser 3, ossification into the third quarter of the crest (50%–75%); Risser 4, ossification into the fourth quarter of the crest (more than 75%) to completion of the apophyseal line excursion; and Risser 5, fusion of the apophyseal ring to the ilium, from the start of the process posterior-medially to its completion [10].

In the *FM* of Risser’s staging, which is widely used in Europe, the iliac crest is divided into three parts, and there are six grades of staging as follows: Risser 0, no ossification; Risser 1, ossification within the first anterior third (33%); Risser 2, ossification extending into the second third (33%–66%); Risser 3, ossification of the entire apophysis (66%–100%); Risser 4, beginning of fusion of the apophysis to the ilium posterior-medially; and Risser 5, complete fusion of the apophysis to the ilium [10].

The Risser staging was performed on a whole spine anterior-posterior AP view using the two traditional methods. While performing the Risser staging using a 3D-CT scan, the widest view of the iliac bone was obtained by rotating the 3D images to evaluate the true excursion ratio between the apophysis and the iliac crest. The iliac crest was divided into four equal sections in the *OM* and into three equal sections in the *FM*. The length of the iliac apophysis ossification was described as a percentage of the total length of the iliac crest. If there was a doubt regarding fusion of the apophysis, it was confirmed on coronal sections.

Additionally, while evaluating ossification of the iliac apophysis on 3D-CT, certain variations in patterns of ossification, excursion, and fusion of the iliac apophysis were observed and were classified as conventional ossification, skip ossification, isolated *non-linear* ossification, micro-fusion, or pseudo-fusion. Conventional ossification was defined as ossification of the iliac crest progressing in a linear pattern from anterior to posterior, while skip ossification was defined as a step (discontinuity) in the ossification of the iliac apophysis. Isolated non-linear ossification was described as only round-shaped, non-linear ossification in the anterior or posterior aspect of the iliac crest, and micro-fusion was defined as- anterolateral or posteromedial fusion visualized on coronal cut or 3D reconstructed CT images, with no fusion seen on plain radiography regardless of apophyseal excursion. Lastly, pseudo-fusion was defined as the presence of a clear sclerotic line at the posterior iliac spine on *PR* but absence of fusion on coronal cuts or 3D reconstructed images of CT.

Two orthopedic surgeons who were blinded to the measurements repeated Risser’s staging three times using OM and FM on *PR* and 3D-CT images at one-week intervals. If there was disagreement over the staging of Risser’s sign, the final Risser stage was determined after a discussion but before the calculation of concordance rates. The data was then reevaluated by an independent radiologist who did not participate in the study. Thereafter, the concordance rate of Risser’s staging between *PR* and 3D-CT was calculated.

### Statistical evaluation

Weighted kappa statistics were used to analyze 3D-CT and *PR* intra- and inter-observer Risser’s staging reliability values and results were expressed as kappa (κ) values. A kappa value greater than 0.81 was considered nearly perfect agreement, 0.61–0.80 as substantial agreement, 0.6–0.41 as moderate agreement, 0.4–0.21 as fair agreement, 0.2–0.01 as slight agreement, and a value below 0.01 as poor agreement [11].

The concordance rate of each Risser’s stage was evaluated using Fisher’s exact test. A *p* value less than 0.05 was considered statistically significant. SPSS 13.0 for Windows (SPSS Inc., IL, USA) was used for the statistical analysis.

## Results

The study included 27 males and 62 females. The mean chronological age was 12.3 ± 5.9 years (range, 10–18 years). The *distribution of enrolled patients according to the method of Risser’s staging is described in Tables*1*and*2*. All Risser’s stage PR and 3D-CT intra- and inter-observer reliability values were greater than 0.9 regardless of the measurement method (Table*3*).*

**Table 1:** **Analysis of concordance between radiography and computed tomography according to Risser’s stage using the Original method**

**Stage determined by CT**	**Stage determined by plain radiography**						
Stage	0	1	2	3	4	5	Total
0	11	1	0	0	0	0	12
1	3	0	2	0	0	0	5
2	0	2	5	0	0	1	8
3	0	0	2	1	1	1	5
4	0	0	1	5	15	14	35
5	0	0	0	0	3	21	24
Total	14	3	10	6	19	37	89
Concordance rate	78.57%	0.00%	50.00%	16.67%	78.95%	56.76%	59.55%

The distribution of error types was as follows: miscalculation of excursion (*n* = 12), skip ossification (*n* = 3), isolated *non-linear* ossification (*n* = 3), micro-fusion (*n* = 3), pseudo-fusion (*n* = 14), and presence of gas shadow (*n* = 1). Further, the results of the statistical analyses revealed a significant difference between stages (*p* = 0.011), with stages 1, 2, 3, and 5 exhibiting a lower rate of concordance (<70%). According to *FM*, concordance rates were 78.57% in stage 0, 40.00% in stage 1, 66.67% in stage 2, 80.95% in stage 3, 36.00% in stage 4, and 100% in stage 5 (Table 2).

*CT* computed tomography, *%* percentage.

**Table 2:** **Analysis of concordance between radiograph and computed tomography according to Risser’s stage determined using the French method**

**Stage determined by CT**	**Stage determined by plain radiography**						
Stage	0	1	2	3	4	5	Total
0	11	1	0	0	0	0	12
1	3	2	1	0	0	0	6
2	0	1	6	0	2	0	9
3	0	1	2	17	10	0	30
4	0	0	0	4	9	0	13
5	0	0	0	0	4	15	19
Total	14	5	9	21	25	15	89
Concordance rate	78.57%	40.00%	66.67%	80.95%	36.00%	100.00%	67.42%

The distribution of different types of error using FM was as follows: miscalculation of excursion (*n* = 4), skip ossification (*n* = 3), isolated *non-linear* ossification (*n* = 2), micro-fusion (*n* = 8), pseudo-fusion (*n* = 10), and gas shadow (*n* = 2). The results of the statistical analyses revealed a significant difference between stages (*p* = 0.0001), with stages 1, 2, and 4 exhibiting a lower rate of concordance (<70%). The mean concordance rate between *PR* and 3D-CT for assessing Risser’s staging was 59.76% for *OM* and 67.42% for *FM*.

*CT* computed tomography, *%* percentage.

**Table 3:** **Reliability of the Original and French methods for Risser’s staging using CT or plain radiography**

	**Inter-observer reliability**	**Intra-observer reliability**
Original method (radiograph)	0.94^a^	0.91^a^
Original method (CT)	0.99^a^	0.98^a^
French method (radiograph)	0.92^a^	0.91^a^
French method (CT)	0.99^a^	0.97^a^
Comparison of concordance rate between radiograph and CT according to Risser’s stages		
Original method	0.000008^b^	
French method	0.10^b^	

*AP* anterior-posterior, *CT* computed tomography.

^a^Weighted kappa statistics were used, and all values are expressed as kappa values.

^b^Fisher’s exact test was used, and all values are expressed as *p* values.

According to the results using *OM*, concordance rates were 78.57% in stage 0, 0.0% in stage 1, 50.00% in stage 2, 16.67% in stage 3, 78.95% in stage 4, and 59.55% in stage 5 (Table 1).

## Discussion

The Risser staging system is widely used to assess the potential for progression of spinal curvature in *AIS* in combination with other predictive factors such as triradiate cartilage closure, mean height velocity, Tanner’s staging, radiographs of hand and elbow, and chronological age [12]. Risser’s staging is the most commonly used method for assessing skeletal maturity because it is easily determined *using pelvic radiography*. Likewise, Risser’s staging is a well-established follow-up tool for determining the acceleration and cessation of vertebral growth in AIS patients and is widely used for research purposes.

However, several reports in the literature have suggested that the Risser staging system is less accurate for predicting vertebral growth than hand-wrist radiography, skeletal age, height, and even chronological age [6,7,12,13]. There are also doubts regarding the reliability and accuracy of Risser’s staging when assessed using only posteroanterior plain radiographs, because ossification of the iliac apophysis is best viewed on anteroposterior radiographs. Izumi et al. reported only 58% agreement between posteroanterior and anteroposterior Risser’s staging [3]. A hypothesis in the current study was that one of the reasons for the low accuracy of Risser’s staging in the determination of spinal growth potential might be inaccurate identification of the Risser stage itself. In the literature, inaccurate staging has been attributed to inability to completely visualize the iliac apophysis due to superimposition of the iliac bone in frontal radiographs and to variations in iliac apophysis excursions, fusions, and ossifications [3-6]. Reports on the frequency of such variations range from 10% to 41%. Risser observed fragmentary development of the iliac apophysis with interruptions that later resolved the appearance of iliac epiphysis at the posterior side (1%) and variable excursions of the apophysis in 10% of cases. Zaoussis and James observed apophysis with short excursions in 10% of cases, early fusion of the apophysis in 24%, and posterior ossification in 40% of cases. In their study of 34 patients, Shuren et al. found five reverse progressions, five cases of capping in fragments, including three cases that started capping toward the middle, and one case of partial fusion during capping. Such anomalous patterns of iliac apophyseal ossification may introduce errors into Risser stage calculations and further decrease the accuracy of *PR* for its calculation.

For the above reasons, some authors have suggested the use of lateral spinal radiographs in addition to *plain pelvic radiographs* to reliably detect iliac apophysis excursions and fusions and demonstrated that the added radiographs improved accuracy [5]. Other authors tried to increase the reliability of Risser’s staging using anteroposterior and posteroanterior bending views but found no significant difference from conventional AP radiographs [6]. However, these studies had inherent limitations in detecting the apophysis ossification and fusion, because evaluations were performed using only *PR*, which is a two-dimensional modality [3-6]. In order to verify the accuracy of *PR* for estimating the Risser’s stage, it is necessary to compare the results of PR with those of other diagnostic tools that have high sensitivity and specificity in detecting ossification and fusion. For this reason, 3D-CT was selected for the current study because the reconstructed images allow accurate visualization of the excursions and fusion of the iliac apophysis. The 3D-CT also has increased sensitivity for detecting different variations in the usual pattern of iliac apophyseal ossifications, fusions, and excursions [7-9].

Variable Risser’s staging inter- and intra-observer reliability values following application of either *OM or FM* have been reported in the literature. Generally, previous studies have indicated good inter- and intra-observer reliability and reproducibility for calculating Risser’s stages [14,15]. Dhar et al. [16] showed an inter-observer agreement of 89.2% and an intra-observer agreement of 93.4%, while Goldberg et al. [2] obtained a kappa value of 0.80, which indicated excellent inter-observer agreement. Conversely, studies by *Hammond et al.* [17] *and Shuren et al.* [6] *revealed only moderate agreement between radiologists and orthopedic surgeons for interpretation of Risser’s stage and reported poor inter-observer reliability (kappa values of 0.31 and 0.53, respectively)*.

The current study demonstrated high intra- and inter-observer reliability between PR and 3D-CT for Risser’s staging using both *OM and FM*. However, the mean concordance rate between radiography and 3D-CT was only 59.55% using *OM* and 67.42% using *FM* (Table 3).

The results suggest that *PR* was less accurate for Risser’s sign staging when verified with 3D-CT. Furthermore, the concordance rates of Risser stages 1, 2, 3, and 5 in *OM* and stages 1, 2, and 4 in *FM* were less than 70%.

Previous studies employed serial *PR* to evaluate iliac apophyseal variations. Knowledge and awareness of these variations minimizes errors while staging. Serial CT evaluations of patients are not recommended, however, and the value of a single 3D-CT study when evaluating the exact nature of iliac apophyseal variations and their frequency is limited. With this in mind, patients with Risser’s staging values from PR and CT that did not match were further analyzed to identify the reasons and sources of the errors. The following common sources of errors that might lead to misinterpretation in Risser’s staging were identified: miscalculation of excursion, isolated *non-linear* ossification, skip ossification, pseudo-fusion, and micro-fusion. The distribution of errors that occurred at each stage while calculating Risser’s grade using OM and FM is presented below. Risser’s stage, as determined by a CT scan, was considered the real stage of the patient.

**Figure 1:**
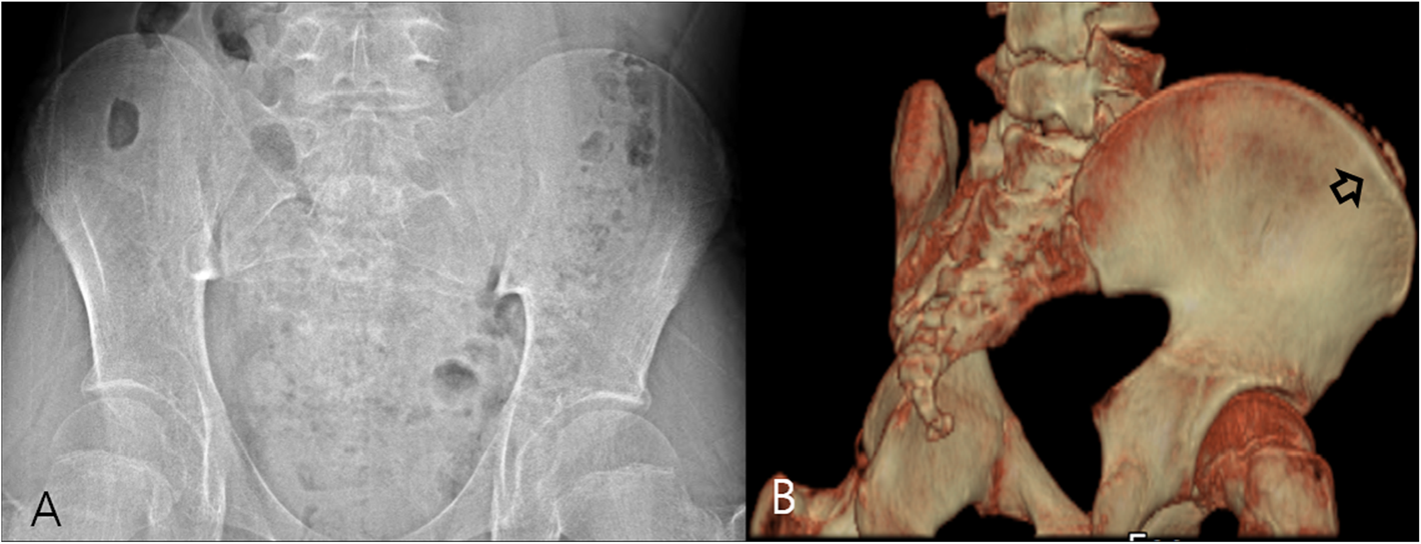
**Micro**-**ossification. (A)** Plain radiography performed on a 12-year-old boy did not reveal excursion of the apophysis and was graded as Risser stage 0. **(B)** A 3D-CT image of the same patient revealed micro-ossification of the iliac apophysis and was graded as Risser stage 1. The lower resolution and sensitivity of PR failed to show isolated non-linear ossification.

**Figure 2:**
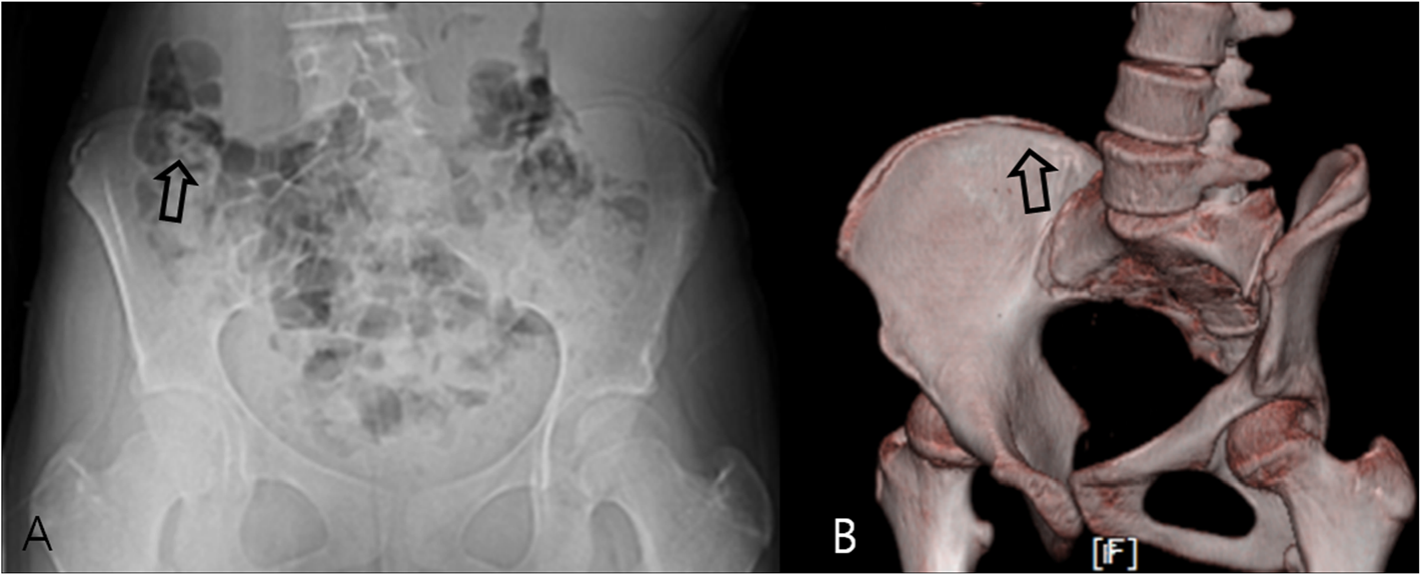
**Gas shadow. (A)** Plain radiography performed on a 13-year-old girl, a gas shadow on the posterior iliac crest prevented interpretation of bilateral excursion of the apophysis, and the patient was graded as Original Risser stage 2 and French Risser stage 1. **(B)** A 3D-CT image of the same patient, excursion of the iliac apophysis was clearly visible along the entire iliac bone, and was graded as Original Risser stage 4 and French Risser stage 3. Gas and internal organ shadows interfered with accurate staging.

**Figure 3:**
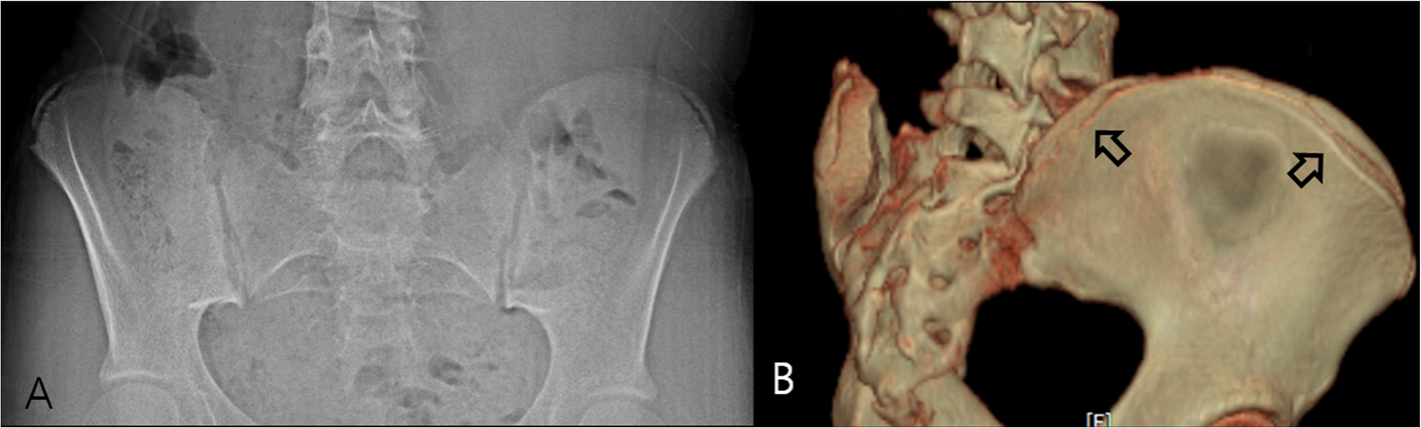
**Skip ossification. (A)** Plain radiography performed on a 14-year-old girl revealed excursion of the apophysis, approximately covering 60% of the iliac bone, and was graded as Original Risser stage 3 and French Risser stage 2. **(B)** A 3D-CT image of the same patient. Showing skipped ossification at the posterior iliac crest with grading of Original Risser stage 4 and French Risser stage 3. Initial apophyseal ossification of the ilium was not linear, but occurred in a skip pattern, which was not visualized properly by plain radiography.

Risser stage 0 *on* PR by both methods was determined to be Risser stage 1 on a CT scan *due to micro-ossifications and gas shadows. The inaccuracy of* Risser stage 1 on PR was *graded differently* on 3D-CT*. The main cause for the discrepancy was* the presence of skip ossification of the iliac apophysis, which led to an underestimation of the Risser’s stage. *Additionally, one* patient had a soft organ shadow that was interpreted as ossification (Table 4, Figures 1, 2, 3).

**Table 4:** **Stage wise distribution of causes of misinterpretations of Risser’s stage**

**Risser’s stage**	**Original method**	**French method**
Stage 0	Micro-ossification (2)^1^ Gas shadow (1)^2^	Micro-ossification (2)^1^ Gas shadow (1)^2^
Stage 1	Skip ossification (2)^3^ Gas shadow (1)^2^	Gas shadow (1)^2^ Skip ossification (2)^3^
Stage 2	Skip ossification (1)^3^ Miscalculation of excursion (4)^4^	Skip ossification (1)^3^ Miscalculation of excursion (2)^4^
Stage 3	Miscalculation of excursion (5)^4^	Micro-fusion (4)^5^
Stage 4	Miscalculation of excursion (1)^4^ Micro-fusion (3)^5^	Skip ossification (2)^3^ Pseudo-fusion (10)^6^Micro-fusion (4)^5^
Stage 5	Skip ossification (2)^3^ Pseudo-fusion (14)^6^	

The numbers inserted in superscripts stand for the figure numbers in which the entities were illustrated.

**Figure 4:**
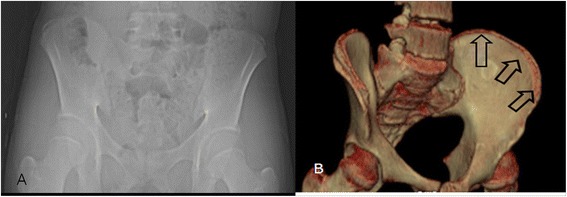
**Inaccurate measurement of excursion. (A)** Plain radiography performed on a 13-year-old girl; the apophysis excursion was visible covering approximately 40% of the iliac bone, and the patient was graded as Original Risser stage 2 and French Risser stage 1. **(B)** A 3D-CT image of the same patient; apophysis excursion was visible up to the posterior superior iliac spine and was graded as Original Risser stage 4 and French Risser stage 3.

**Figure 5:**
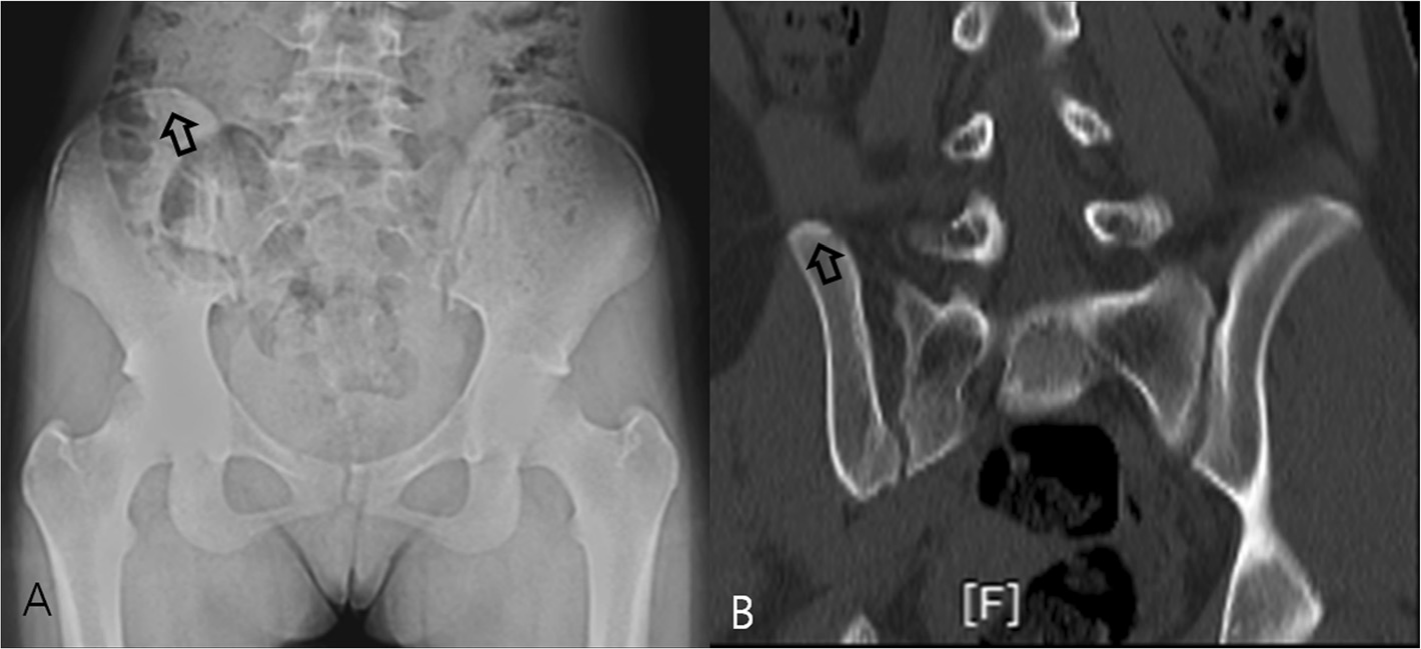
**Micro**-**fusion. (A)** Plain radiography of a 14-year-old girl; a radiolucent line was visible between the apophysis and the posteromedial iliac bone and was graded as Original Risser stage 4 and French Risser stage 3. **(B)** A coronal 3D reconstruction view; there was absence of any radiolucent line indicating definite fusion, and the staging by Original method was 5 and by French method was 4. Accurate estimation of fusion of iliac apophysis or micro-fusion is difficult on plain radiography.

**Figure 6:**
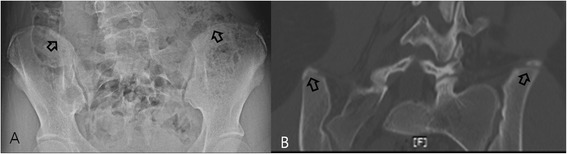
**Pseudo**-**fusion. (A)** Plain radiography of a 15-year-old girl; a sclerotic line was visible on the posterior iliac crest suggesting fusion of the iliac apophysis, and the patient was graded as Original Risser stage 5 and French Risser stage 4. **(B)** A coronal 3D reconstruction view; there was no definite fusion of the apophysis , and the patient was graded as Original Risser stage 4 and French Risser stage 3. Estimating the initiation of pseudo-fusion is difficult on plain radiography.

For Risser stage 3 determined using PR, the main cause of error was different in OM and FM, which reflected the difference in the two staging systems. In *OM*, stage 3 is the anterior to posterior changing point for the excursion direction, whereas in *FM*, stage 3 is defined as full excursion of the apophysis without fusion. *Hence, the most common cause of error in OM was miscalculation of excursion (Figure*4*), whereas difficulty detecting micro-fusion was the most frequent source of error in FM*. In Risser stages 4 and 5 determined using PR, the main cause of error was again different for OM and FM and was due to disparities in the staging systems. For OM, the main cause of error resulted from *the inability to detect* micro-fusion in stage 4 and pseudo-fusion in stage 5 (Table 4). However, the main cause of FM stage 4 errors was pseudo-fusion, whereas stage 5 exhibited perfect concordance between radiography and 3D-CT. In many cases, despite the observation of *only* a lucent posteromedial physeal line on PR, 3D-CT clearly demonstrated micro-fusion (Figure 5A,B). Meanwhile, in cases of a clear posteromedial sclerotic line, which suggested fusion on radiographs, 3D-CT demonstrated a clear lack of fusion on coronal cuts (i.e., pseudo-fusion; Figure 6A,B). Errors in both micro-fusion and pseudo-fusion occurred because PR lacks the level of sensitivity required for the accurate detection of iliac apophysis ossification and fusion. In addition, pelvic rotation, which is present in mild to moderate scoliosis deformities, adds to the spatial distortion. When analyzing *OM and FM* concordance rates according to their stage, the concordance rates of *FM* were more constant than the resulting *OM rates*. A possible explanation for the divergent concordance rates might be the difference in the division of the iliac apophysis employed by each of the two methods (division into four portions in *OM* and into three portions in *FM*). Errors caused by low resolution and the subsequent reduced capacity of PR to detect fusions, including pseudo-fusions and micro-fusions, were greater in stage 5 using OM and in stage 4 using FM. These stages signify the early fusion period during which fusion seems to proceed from pseudo-fusion to micro-fusion to complete fusion. FM, which divides fusion into two stages, had a 100% concordance rate in stage 5.

Most of the potential sources of errors can be eliminated. Gas shadows can be easily eliminated by adequate bowel preparation before X-ray acquisition. Obtaining high quality X-rays and observing them against bright light will minimize the failure to detect micro-calcifications [18]. As suggested by some authors, ultrasonography can be a useful tool and can complement X-rays in the detection of micro-calcifications and the actual extent of ossifications and fusions without exposing patients to the risks associated with radiation [19,20]. The addition of extra lateral or oblique views, and considering every available, view will reveal some of the missed excursions of the apophyseal ossification and the actual extent of fusion [4,5]. The modification of Risser’s staging to minimize the divisions of iliac apophysis might lead to a decrease in miscalculation of the apophyseal excursions. Further, increased knowledge and awareness of the common variations among scoliosis patients will help surgeons identify anomalous ossification patterns such as skipped ossification and the appearance of the first posterior-medial ossification and will enable them to appropriately stage cases. Since fusion appears to start as micro-fusion or pseudo-fusion and then proceed to full fusion, fusion-related errors can be minimized by dividing the fusion stages into two categories, similar to FM, and by merging the posteromedial ossification stage with the first fusion stage.

Accordingly, we propose the following modified version of Risser’s staging:

- 0- No ossification.
- 1- Appearance of ossification anywhere, in less than one-third of the iliac wing.
- 2- Ossification of more than one-third and equal to or less than two-thirds of the iliac wing without evidence of any fusion.
- 3- Ossification beyond two-thirds of the iliac wing and appearance of fusion
- 4- Fusion of more than one-third of the iliac crest.
- 5- Complete fusion.

*Because 3D-CT results in exposure to radiation and has relatively high associated costs, PR was used to measure Risser’s stage. However, a limitation of this study was that the new Risser’s staging system was not objectively verified as a viable replacement for OM or FM. However, the inaccuracy of PR used in Risser’s staging system was verified, using a different X-ray device. Moreover, the factors that caused measurement errors were analyzed, and a feasible solution was suggested. Considering that the verification of a new Risser’s staging system requires collection of data and observational time, the presentation of a Risser’s staging system using objective data would be meaningful.*

We also acknowledge a limitation of this study was the uneven distribution of the enrolled patients according to Risser’s stage, but we were careful not to overestimate or underestimate the results, and to indicate only the overall patterns. For this reason, we merely listed the main causes of error and did not perform detailed statistical analysis.

## Conclusions

*PR* for Risser’s staging is reliable but is not a suitable tool for accurate measurement of the excursion of apophyseal ossification. *PR* has limitations in determining the actual lengths of apophyseal ossification and fusion. We have proposed various guidelines to minimize errors and to increase the accuracy of Risser stage determination by *PR*, together with modifications to the Risser staging system.

### Ethical review committee statement

The authors certify that this study involving human subjects is in accordance with the Helsinki declaration of 1975 as revised in 2000, and that the relevant institutional ethical committee approved the study.
